# DOES ASSET POVERTY INFLUENCE HEALTH AND WELL-BEING IN LATER LIFE? AN 8-YEAR PROSPECTIVE STUDY IN CHINA

**DOI:** 10.1093/geroni/igae098.2505

**Published:** 2024-12-31

**Authors:** Yu-Chih Chen, Ting Hu

**Affiliations:** The University of Hong Kong, Hong Kong, China (People’s Republic); University of Illinois Urbana-Champaign, United States

## Abstract

Wealth serves as a financial cushion for older adults that can be consumed to generate economic, psychological, and social benefits in later life. Asset poverty, defined as inadequate wealth to cover basic needs for three months, can lead to financial instability and diminished well-being. However, research on its impact on the health and well-being of older adults, particularly in developing countries like China, is scarce. Using data from 7,982 adults aged 60 and older from the 2011-2018 China Health and Retirement Longitudinal Study, this study investigates the effects of asset poverty on cognition, self-rated health, depressive symptoms, and life satisfaction. Four types of asset poverty across time (i.e., persistent asset poverty, transitioning into asset poverty, transitioning from asset poverty, and persistent asset non-poverty) were constructed using three varied wealth measures: total assets, financial assets, and non-housing assets. Findings from the two-way fixed effect model showed that, compared to those consistently above the asset poverty line, households falling into asset poverty or experiencing prolonged asset poverty reported lower self-rated health and life satisfaction, along with higher depressive symptoms. Even transitioning out of asset poverty correlated with lower self-rated health and life satisfaction. However, asset poverty was not associated with cognition. This study underscores the significance of maintaining adequate assets to guard against financial insecurity and diminished mental health. Policies aimed at fostering lifelong asset accumulation and development should be advocated to bolster health and well-being in later life.

